# Mesenchymal stem cells in tissue repairing and regeneration: Progress and future

**DOI:** 10.4103/2321-3868.113330

**Published:** 2015-06-26

**Authors:** Jiafei Xi, Xinlong Yan, Junnian Zhou, Wen Yue, Xuetao Pei

**Affiliations:** Stem Cell and Regenerative Medicine Lab, Beijing Institute of Transfusion Medicine, 27, Tai Ping Road, Beijing, 100850 China

**Keywords:** Mesenchymal stem cells, regenerative medicine, gene therapy, anticancer, immune disorder

## Abstract

The presence of mesenchymal progenitor cells within bone marrow has been known since the late nineteenth century. To date, mesenchymal stem cells (MSCs) have been isolated from several different connective tissues, such as adipose tissue, muscle, placenta, umbilical cord matrix, blood, liver, and dental pulp. Bone marrow, however, is still one of the major sources of MSCs for preclinical and clinical research. MSCs were first evaluated for regenerative applications and have since been shown to directly influence the immune system and to promote neovascularization of ischemic tissues. These observations have prompted a new era of MSC transplantation as a treatment for various diseases. In this review, we summarize the important studies that have investigated the use of MSCs as a therapeutic agent for regenerative medicine, immune disorders, cancer, and gene therapy. Furthermore, we discuss the mechanisms involved in MSC-based therapies and clinical-grade MSC manufacturing.

## Introduction

Knowledge of the presence of mesenchymal progenitor cells within bone marrow can be traced back to the late nineteenth century to the work of Goujon[1] and Biakow[2] on the heterotopic transplantation of rabbit marrow. The definitive evidence that bone marrow includes some non-hematopoietic, plastic-adherent precursor cells was originally provided by Friedenstein and his colleagues, who grew whole bone marrow cells in a medium supplemented with 10% selected fetal bovine serum to demonstrate the ability of a rare population of plastic-adherent cells (approximately 1 in 10,000 nucleated cells in the bone marrow) to form spindlelike and round-shaped colonies. Friedenstein defined these fibroblast precursors as fibroblast colony-forming units (CFU-F), analogous to hematopoietic colony-forming units.[3] As other groups subsequently extended these initial observations, the CFU-F progenitors were accepted to be multipotent and able to differentiate into adipocytes, osteoblasts, and chondrocytes. Arnold Caplan first used the term mesenchymal stem cells[4] based on the notion of a stromal stem cell proposed by Maureen Owen.[5] To date, mesenchymal stem cells (MSCs) have been reported to have been isolated from several different connective tissues, such as adipose tissue, muscle, placenta, umbilical cord matrix, blood, liver, and dental pulp. Bone marrow, however, is still one of the major sources for MSCs used in preclinical and clinical research.[6]

**Table 1:** 

Access this article online	
**Quick Response Code**:	**Website**: www.burnstrauma.com
	**DOI**: 10.4103/2321-3868.113330

Since the initial use of the term “MSC,” the stem cell identity of MSCs has been the subject of considerable debate. In 2000, a workshop at the Annual Meeting of the International Society for Cellular Therapy (ISCT) concluded that there was a lack of solid data in support of the ‘stemness’ of unfractionated plastic-adherent cells from bone marrow.[7] In 2005, the ISCT proposed “multipotent mesenchymal stromal cells” as a replacement term for “mesenchymal stem cells,” although the two terms shared the abbreviation “MSC”.[8] The ISCT then suggested minimal criteria for the definition of human MSCs: (1) MSCs must be plastic-adherent when maintained in standard culture conditions; (2) MSCs must express CD105, CD73, and CD90, while lacking expression of CD45, CD34, CD14 or CD11b, CD79a or CD19, and HLA-DR surface molecules; and (3) MSCs must be capable of differentiating into osteoblasts, adipocytes, and chondroblasts *in* vitro.[9] All criteria must be satisfied, as no single characteristic is sufficient for identifying MSCs. The bone marrow-derived plastic-adherent cell population also contains endothelial cells, fibroblasts, and macrophages. Contamination by hematopoietic and endothelial cells can be ruled out by the combination of cell surface markers. However, no specific markers currently exist that can reliably discriminate between passaged MSCs and fibroblasts. Furthermore, colony-forming capacity and differentiation potential are important specific properties that distinguish MSCs from fibroblasts.[10] Recently, it was reported that the level of expression of CD166 was significantly higher in MSCs than in fibroblasts, while the expression level of CD9 was significantly lower. CD146 was found to be exclusively expressed in MSCs; however, CD146 was downregulated and CD9 was upregulated with the passage of MSCs. The expression levels of all other markers were unchanged.[11]

Initially heralded as stem cells, MSCs were first evaluated for regenerative applications. MSCs have since been shown to directly influence the immune system[12] and to promote the neovascularization of ischemic tissues.[13,14] These observations have prompted MSC transplantation as a treatment for various diseases. In this review, we summarize the important studies of MSCs that describe the potential use of these cells as a therapeutic agent for regenerative medicine, immune disorders, cancer, and gene therapy. Furthermore, we discuss the mechanisms involved in MSC therapy as well as clinical-grade cell manufacturing of MSCs.

## Identification of mesenchymal stem cells

Although MSCs have been isolated from many postnatal organs and tissues, bone marrow stroma is still the most recurrent tissue source utilized in cultivating MSCs. Most MSC populations have been isolated using methods similar to those originally used by Friedenstein and his colleagues.[15] In general, low-density mononuclear cells from normal human donors are plated in a basal medium supplemented with selected batches of fetal bovine serum, and the cells that readily adhere to plastic culture dishes and form large CFU-F clones are considered to be primary MSCs. Although endothelial cells, macrophages, lymphocytes, and differentiated smooth muscle cells can also adhere to plastic and contaminate the MSC culture, these cells are not successfully passaged and expanded in the specialized culture medium. After a few passages, the MSC cultures display a rather homogenous population of fibroblast-like cells. These cells lack CD11 and CD14 (monocyte and macrophage markers), CD34 (primitive HSCs and endothelial cells), CD45 (pan-leukocytes), CD19 (B cells), CD3 (T-cell receptor), CD31 (endothelial cells), and HLA-DR, but they show expression of CD29, CD144, CD166, CD105, and CD90. All of these markers are used retrospectively to identify MSCs via the elimination of endothelial and hematopoietic cell contaminants.[16] Immunofluorescence and immunohistochemical staining have also demonstrated that bone marrow (BM)-MSCs were positive for myofibroblastic markers, such as α-SMA, vimentin, fibronectin, and N-cadherin, but negative for epithelial markers, such as CK18 and E-cadherin.

Until now, the best marker for prospectively identifying MSCs has remained unclear. A few papers have reported the isolation of MSCs using surface markers such as Nestin,[17] stage specific embryonic antigen-1 (SSEA-1),[18] SSEA-4,[19] and Stro-1. Nestin-positive cells in human bone marrow represent true mesenchymal stem cells in that they show a close physical association with hematopoietic stem cells (HSCs) and can express a high level of core HSC maintenance genes. However, whether this putative MSC marker can be utilized to isolate MSCs from other tissues remains to be determined.

In addition to the morphologic and phenotypic characteristics of MSCs, one of the hallmarks for the identification of MSCs is their multipotent ability to differentiate into various lineages, including adipose, bone, cartilage, and myogenic cells.[9] To induce adipogenic differentiation, MSCs were cultured in regular medium supplemented with dexamethasone, insulin, isobutyl methyl xanthine, and indomethacin. Adipogenic differentiation was verified by the accumulation of lipid vacuoles, which were detected by Oil Red O staining. These differentiated cells also expressed adipogenic lineage-specific genes, such as peroxisome proliferation-activated receptor γ2 (PPARγ2), lipoprotein lipase (LPL), fatty acid-binding protein (aP2), adipsin, and leptin. To promote the differentiation of MSCs into osteoblasts, confluent monolayers of MSCs were incubated with ascorbic acid, ß-glycerol phosphate, and dexamethasone; in addition, bone morphogenic proteins (BMPs), particularly BMP-2 and BMP-6, were sometimes added to the induction system and strongly promoted osteogenesis in MSCs. Early osteogenic differentiation was evaluated by alkaline phosphatase staining, and late osteogenic differentiation was assessed by von Kossa staining for calcium mineralization detection. Meanwhile, specific gene expression of osteocalcin (OCN), osteopontin (OPN), Runx2, and alkaline phosphatase (ALP) was determined to confirm osteoblastic differentiation. For chondrogenic differentiation, MSCs were usually centrifuged gently to form a micromass and then cultured in serum-free High Glucose Dulbecco’s Modified Eagle Media (HG-DMEM) and supplemented with dexamethasone, Insulin-Transferrin- Selenium (ITS), ascorbic acid, sodium pyruvate, proline, and transforming growth factor-ß3. The development and accumulation of cartilage matrix was shown by staining proteoglycans with toluidine blue, alcian blue, and safranin O. Differentiated MSCs also express chondrogenic-specific genes, such as type II collagen and aggrecan.[20] Further studies have shown that MSCs can also differentiate into bone marrow stromal cells, tendon-ligament fibroblasts, myocytes, endothelial cells, neurons, and tenocytes.[21]

## Mesenchymal stem cells for regenerative medicine

**Figure 1:**
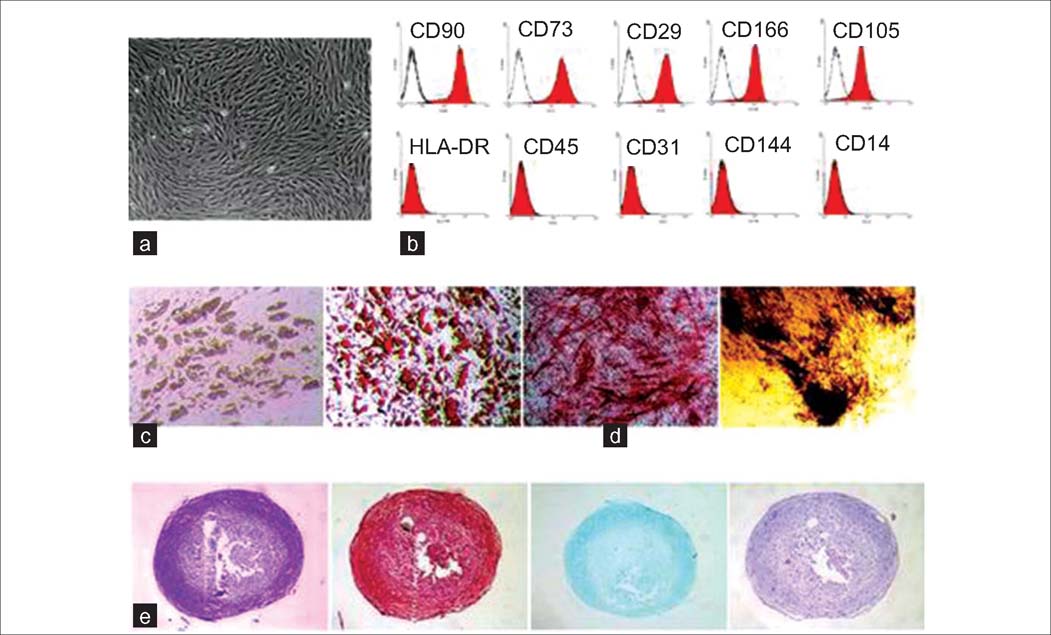
Characteristics of MSCs from adult healthy bone marrow (Modified from Yan *et al.*[20]) (a) Representative morphology of MSCs derived from healthy adult bone marrow. (b) Flow cytometry analysis of BM-MSC showed that BM-MSC is positive for mesenchymal markers and negative for endothelial and hematopoietic markers. (c) After specific adipogenic induction, BM-MSCs showed many lipid vacuoles that were verified by Oil Red O staining. (d) After being cultured in an osteogenic medium for 4 weeks, BM-MSCs showed early osteogenic differentiation capacity through ALP staining. Late calcium deposits in the extracellular matrix were verified by von Kossa staining. (e) BM-MSCs demonstrated chondrogenic differentiation potential as shown by toluidine blue staining, Alcian blue staining, Safranin O staining and H&E staining (cited from Fiorina *et al*).

It is known that MSCs can be used for the treatment of injured myocardium, skin, pancreas, and bone. Although the exact mechanisms of action have not been fully elucidated, studies show that MSCs can act on several levels of endogenous repair to bring about treatment of disease. MSCs have been shown to directly promote tissue repair.[22,23] When administered to treat animals undergoing acute renal failure, MSCs promoted the proliferation of renal-tubule epithelial cells.[24] MSCs can directly protect and repair damaged ß-islets from autoimmune attacks [Figure 1].[25] MSCs can differentiate into osteoblasts and chondrocytes. The osteoblastic potential of MSCs may be exploited for treating long bone defect.[26] Although MSCs are effective for long bone reconstruction, MSCs are not effective for jaw reconstruction[27] because MSCs from the bone marrow cannot differentiate into osteoblasts when implanted in the jaw[28] These data emphasize the importance of the origin of MSCs used for tissue repair.

Evidence for the actual and potential clinical applications of MSCs for cardiovascular diseases comes from the findings that MSCs can spontaneously differentiate into beating cardiomyocytes that respond to physiological and pharmacological stimuli.[29] Many clinical trials have been performed that primarily focused on treating heart damage.[30] The treatment was found to be safe and resulted in improved cardiac performance of the MSC-injected group and is thus very encouraging.

It was shown that MSCs could improve the healing of skin defects in humans. Recent data suggest that autologous and allogeneic MSCs appear to be equally effective for wound repair.[31,32] A shortcoming of using MSCs for wound repair is the heterogeneity of methods used for MSC culture as well as the lack of functional characterization of MSCs.

Finally, MSCs have also been proposed for the treatment of spinal cord injury,[33] stroke,[34] amyotrophic lateral sclerosis[35] and neurometabolic diseases.[36]

## Mesenchymal stem cells for treating immune disorders

MSCs demonstrate a profound inhibitory effect on T, B, dendritic cells and natural killer cell proliferation *in vitro* and *in vivo*. These results suggested that MSCs could be used to cure immune disorders. Bartholomew *et al.*,[37] first demonstrated that the administration of MSCs could prolong skin graft survival. Currently, more than 12 preclinical animal models have been developed to demonstrate the capabilities of MSCs in modulating the immune system.[38] Le Blanc and colleagues have shown that MSCs can successfully cure severe, treatment-resistant graft-versus-host disease (GvHD).[39,40] However, the use of MSCs to prevent the rejection of allogeneic organ transplantation is still limited to animal models, and the obtained results have been conflicting.[41]

MSCs have also been proposed as a treatment for autoimmune diseases.[42] The intravenous injection of MSCs into diabetic nonobese diabetic/severe combined immunodeficiency (NOD/SCID) mice resulted in an increased number of pancreatic islets and insulin-producing ß cells.[43] Indeed, MSCs have been used for the treatment of rheumatoid arthritis (RA), systemic lupus erythematosus (SLE) and multiple sclerosis (MS).

Studies have been performed to investigate the effect of MSCs on experimental autoimmune encephalomyelitis (EAE).[44] More recently, it was shown that the intravenous infusion of MSCs also suppresses pathogenic B cell responses *in vivo*.[45] A decrease in neuronal loss was observed in EAE mice treated with MSCs, suggesting a protective effect on damaged tissues.[45]

MSCs can also provide support for the growth and differentiation of haemopoietic progenitor cells and can promote the engraftment of haemopoietic stem cells. It was shown that the proliferation of activated lymphocytes were suppressed by MSCs in a non-human leukocyte antigen (HLA) restricted manner.[46] A clinical trial has been in progress that investigates the use of MSCs in treating hematologic malignancy by co-transplanting MSCs and HSCs from HLA-identical siblings of the study participants.[47] It was exciting that, in 2008, the US Food and Drug Administration (FDA) gave the green light to clinical trials for the application of MSCs in MS and articular cartilage repair.

## Mesenchymal stem cells for anticancer treatment

human MSCs (hMSCs) engraft and remain detectable at the injured sites and can co-localize with the sites of tumor development.[48] Thus, MSCs could be used as drug carriers for the treatment of tumors. The homing potential of hMSCs was demonstrated *in vitro* by hMSC coculture with glioma cells or glioma-conditioned medium that increased hMSC invasiveness.[49] *In vivo*, hMSCs could efficiently home to brain tumors and human glioma xenografts.[50] Unfortunately, culture-expanded hMSCs lose CXCR4, which is a key receptor responsible for hematopoietic stem cell homing.[51] However, *in vitro* 3-D culturing of hMSCs as spheroids increases stromal cell-derived factor 1 (SDF1) signaling, restoring the functional expression of CXCR4 that is crucial for the therapeutic application of MSCs.[51] Many cytokines and growth factors also enhance hMSC migration,[50,52] and many of those factors are secreted by tumor cells.[50] The migration of MSCs toward tumor cells is accompanied by the expression of various proteins.[52] This is similar to the *in vivo* myocardial injury recruitment of MSCs by the establishment of an SDF1 gradient toward the heart.[53]

It was shown that *in vitro* hMSC chondrogenic predifferentiation increases engraftment during *in vivo* cartilage repair.[54] Moreover, both the sustained delivery of growth factors to injected MSCs and the cotransplantation of MSCs with healthy tissue parts[55] can improve the MSC engraftment.[54]

Because of the ability of hMSCs to migrate to distant tumors, strategies of using them in anticancer therapies are being developed. The limited lifespan of hMSCs can be prolonged by gene modification.[56] The tumor suppressors are not downregulated in transduced MSCs, suggesting that the growth of these cells is still normal.[56] It will be very exciting to see if genetically modified MSCs can directly act as a drug vehicle that causes the complete, irreversible elimination of tumors. The treatment of human colon cancer with MSCs expressing a suicidal tyrosine kinase gene resulted in tumor elimination.[57] Therefore, gene-modified MSCs with homing potential could be used to treat tumors that cannot be cured with conventional drugs.

## Mesenchymal stem cells for gene therapy

Genetic engineering of cells has become a mainstay in cell and molecular biology, and MSCs offer a unique opportunity to establish transplantation schemes to correct genetic diseases. These cells may be easily obtained, manipulated genetically, and expanded in number before reintroduction. This eliminates the limitations and risks associated with the delivery of genetic repair material directly to the patient via pathogen-associated vectors. Furthermore, there is far less concern of inappropriate differentiation than in human embryonic stem cells.

If a short-lived effect, such as bone regeneration, is the goal, transient transduction would be the desired outcome and utilized methods might include electroporation, chemical methods, plasmids, and viral constructs such as adenovirus. For the treatment of recessive diseases, permanent transduction is required, which has depended on the use of adeno-associated viruses, retroviruses, and lentiviruses.

However, some hurdles exist that must be overcome before this technology will become practical. The first hurdle is the optimization of *ex vivo* transfection; the second hurdle relates to the durability of the desired gene expression because, in most instances, it has been reported that expression decreases with time. The efficacy of gene therapy using MSCs will depend on the efficiency with which the reagents are incorporated into MSCs and the selection of specific targets.

## Potential mechanisms of mesenchymal stem cell therapy

**Figure 2:**
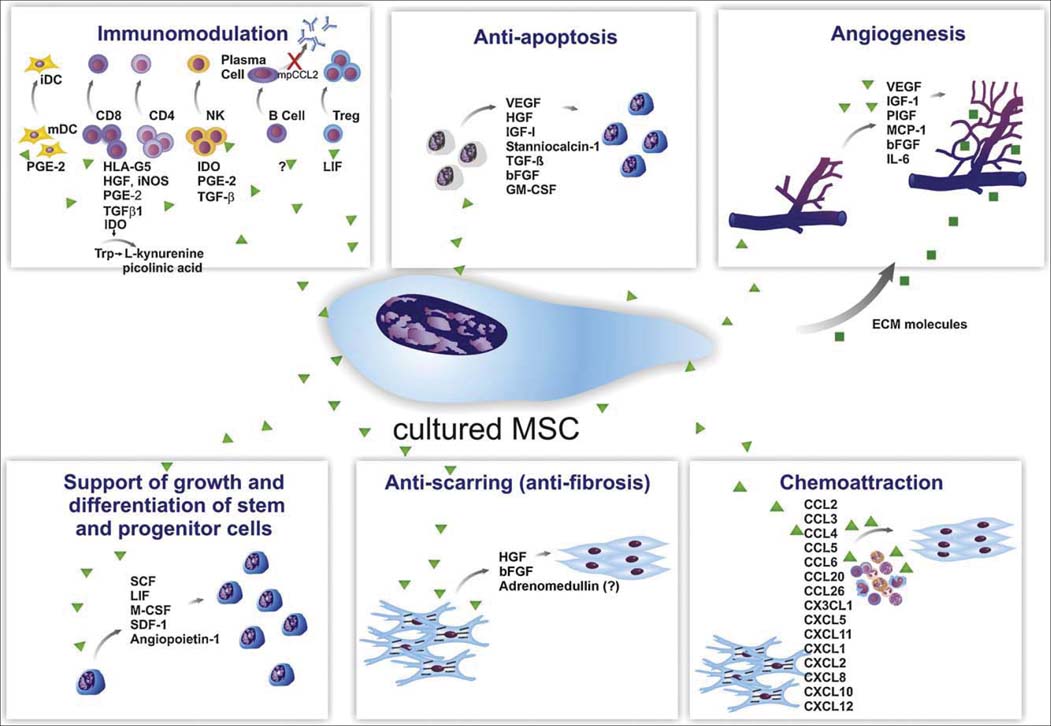
Paracrine effects of cultured MSCs (Cited from da Silva Meirelles *et al.*[59]).

It was once believed that MSCs repair damaged tissues by robustly replacing the damaged cells. However, researchers have shown that in response to injury, MSCs secrete large quantities of bioactive molecules; this constitutes the most biologically significant role of MSCs under injury conditions.[58] We now know that the paracrine effect of MSCs and cell replacement by MSC-derived cells are both important. Understanding this intricate activity as well as the properties of MSCs *in vivo* is central to harnessing their clinical potential [Figure 2].[59]

The secretion of bioactive molecules can be divided into six main categories: immunomodulation, anti-apoptosis, angiogenesis, support of the growth and differentiation of local stem and progenitor cells, anti-scarring and chemoattraction.[59] The number of molecules known to mediate the paracrine action of MSCs increases every day. The immunomodulatory effects of MSCs consist of the inhibition of immune cell proliferation, the suppression of immunoglobulin production, the inhibition of dentric cell maturation and the stimulation of regulatory T-cell proliferation. MSCs also limit apoptosis and stimulate local angiogenesis through the secretion of extracellular matrix molecules. A group of at least 15 chemokines produced by MSCs can elicit leukocyte migration to the injured area.[56]

## Clinical-grade cell manufacture

During clinical applications of MSCs, the culture conditions must be tested and quality controls must be adapted. The release criteria of the cell batch must be strict and must take into account the effectiveness of the cellular product and the safety of the patient. Currently, the MSCs of many clinical research protocols are being generated in hematopoietic cell transplantation (HCT) processing laboratories or similar facilities. Other protocols employ current Good Manufacturing Practice (cGMP) standards, in which the cells are manufactured under the highest standards of sterility, quality control and documentation. A number of factors in the cell manufacturing process influence the nature and function of the MSCs.

Although the ISCT has established the definition of MSCs,[8,9] a major challenge in establishing release criteria is the lack of an accepted functional assay. However, given the wide range of the potential clinical effects of MSCs, any such assays will need to be specific to the particular indication or clinical trial. There may also be significant differences among MSC products from different tissue sources. Researchers have compared adipose-derived MSCs to MSCs from bone marrow,[60] and the source of MSCs may influence the ability of the cells to differentiate. Cell-manufacturing protocols must therefore take into account the variability in the characteristics of the MSCs. To mitigate the malignant transformation of human MSCs, the MSCs to be used for clinical treatment must have undergone fewer than 25–30 cell doublings.[61] The strategy to make the MSC product available quickly is to rapidly grow the MSCs in bioreactors.

## Future perspective

Although perhaps several thousand patients have been treated with MSCs to date, no infusional toxicity or immediate adverse outcomes have been reported, suggesting MSC infusion to be safe. However, MSC therapies have potential risks to patients. The collection of data on patients treated with MSCs and the evaluation of long-term patient outcomes can provide an excellent infrastructure that can avoid publication bias. A registry specific for novel cellular therapies has already been established in the European Group for Blood and Marrow Transplantation (EBMT).[62]

Although many studies have demonstrated the safety and effectiveness of MSC treatment for various diseases, there are still many questions to be answered. The first is to confirm the therapeutic efficacy of transplanted MSCs. The second is to deeply understand the mechanisms of engraftment, homing and *in vivo* differentiation. The third is the availability of validated *in vitro* methods for the large-scale production of MSCs for clinical applications. The final question is the need for novel engineered devices for the tissue-specific delivery of MSCs. As these areas are addressed, the clinical significance of MSCs will be tremendously promoted.
